# Pathogen Behaviour and Survival Dynamics of *Salmonella* Typhimurium, *Escherichia coli* O157:H7 and *Listeria monocytogenes* During the Ripening of ‘Nduja, a Traditional Spreadable Fermented Sausage

**DOI:** 10.3390/pathogens15060606

**Published:** 2026-06-05

**Authors:** Salvatore Pennisi, Luca Nalbone, Mattia Pino, Filippa Lamberta, Graziella Ziino, Alessandro Giuffrida

**Affiliations:** 1 Department of Veterinary Sciences, University of Messina, Viale Giovanni Palatucci 1, 98168 Messina, Italy; pennisisalvatore.21@gmail.com (S.P.); graziella.ziino@unime.it (G.Z.); alessandro.giuffrida@unime.it (A.G.); 2 Riconnexia srls, Spin-Off of the University of Messina, Department of Veterinary Sciences, University of Messina, Viale Giovanni Palatucci 1, 98168 Messina, Italy

**Keywords:** Nduja, challenge test, lactic acid bacteria, fermented meat product, predictive microbiology, non-linear models

## Abstract

This study investigated the behavior of *Salmonella* Typhimurium ATCC 14028, *Escherichia coli* O157:H7 and *Listeria monocytogenes* ATCC 13932 during the ripening of ‘Nduja, a traditional spreadable fermented sausage for which quantitative microbiological data remain limited. An experimental challenge test was conducted under pilot-plant conditions simulating artisanal production, with products inoculated with the three pathogens and monitored over a 28-day ripening period. Microbiological analyses were performed at defined time points, alongside pH and water activity measurements, and inactivation kinetics were modelled using linear and non-linear approaches. The results showed a pathogen-dependent response to the combined antimicrobial hurdles of the process. *Salmonella* Typhimurium was declined to levels below the detection limit by day 28 (–7.10 log CFU/g), while *E. coli* O157:H7 showed a progressive reduction (–3.61 log CFU/g) but persisted at detectable levels. *L. monocytogenes* exhibited the highest resistance, with only a limited reduction (–1.32 log CFU/g); however, no net growth was observed throughout the ripening period, indicating that the product environment did not support its growth. The ripening process was characterized by decreasing pH and water activity, driven by lactic acid bacteria growth, with no differences between inoculated and control samples. Non-linear models provided the best fit to the survival data, highlighting the presence of resistant subpopulations. Overall, the results suggest that ‘Nduja ripening creates conditions unfavorable for sustained pathogen proliferation, although the extent of microbial reduction differed among the investigated microorganisms. These findings provide useful data for the microbiological characterization of this traditional product and may support future risk assessment and process validation studies.

## 1. Introduction

In Europe, traditional agri-food products have gained increasing recognition over recent decades, driven by growing consumer demand for authentic, heritage-based foods and supported by European Union quality schemes such as Protected Designation of Origin (PDO) and Protected Geographical Indication (PGI) [1]. In parallel, considerable scientific attention has been devoted to these products, particularly regarding their quality attributes and hygienic-sanitary characteristics [2]. The resulting body of scientific evidence supports certification processes and compliance with European food safety legislation, providing a scientific basis of value to food business operators and competent authorities alike for ensuring appropriate production practices and effective official control activities [3].

Among ready-to-eat foods, traditional fermented meat products have been extensively investigated with respect to the occurrence and behavior of major foodborne pathogens, yielding essential data for risk assessment and management [4]. However, findings obtained for conventional dry-fermented sausages are not always directly applicable to products that differ substantially in formulation, composition, and processing technology.

This is notably the case of ‘Nduja, a traditional Calabrian (Italy) spreadable fermented sausage currently undergoing efforts towards PGI recognition, particularly in the Spilinga production area, underscoring both its cultural significance and economic relevance, as well as the need for its comprehensive scientific characterization [5]. The manufacturing process is characterized by a distinctive raw material composition, consisting of approximately 50% adipose tissue, 25% lean muscle and 25% chili pepper. Following grinding, sodium chloride (3% *w*/*w*) is added, and the mixture is thoroughly blended before being stuffed into natural pork casings (large intestine and cecum). Ripening, sometimes combined with a smoking step, is carried out for variable durations, ranging from approximately 25 days for pieces of around 500 g to 90 days for pieces exceeding 1 kg [5]. The fermentation process is primarily driven by lactic acid bacteria (LAB), which promotes acidification of the product matrix, competitively inhibits the growth of spoilage and pathogenic microorganisms, and contributes to the development of characteristic organoleptic properties through their metabolic activity [4].

The peculiar physicochemical profile of ‘Nduja, resulting from its high lipid content, elevated chili pepper concentration, and relatively low lean meat fraction, gives rise to microbiological and biochemical dynamics that differ substantially from those of conventional dry-fermented sausages. In particular, the high lipid content contributes to reduced water activity (a_w_) and increased susceptibility to oxidative phenomena, while the artisanal nature of its production may introduce process variability and potential hygienic risks. More broadly, artisanal food production is frequently associated with practices rooted in empirical knowledge and traditional beliefs that are not always aligned with current scientific evidence [6]. This may result in an underestimation of microbiological hazards, potentially exposing consumers to foodborne pathogens. In this context, the limited body of literature available on ‘Nduja has already highlighted the possible presence of pathogenic microorganisms, including *Listeria monocytogenes*, underscoring the need for a deeper characterization of pathogen behavior within this specific food matrix [5,7].

From an epidemiological perspective, recent surveillance data from the European Food Safety Authority (EFSA) and the European Centre for Disease Prevention and Control (ECDC) indicate that meat and meat products, particularly those of porcine origin, rank among the food categories most frequently implicated in strong-evidence foodborne outbreaks in Europe [8]. Furthermore, in 2024, the most commonly reported zoonoses in humans included salmonellosis (79,703 confirmed cases), Shiga toxin-producing *Escherichia coli* (STEC) infections (11,738 confirmed cases), and listeriosis (3041 confirmed invasive cases). The causative agents of these infections, *Salmonella* spp., STEC, and *L. monocytogenes*, represent relevant biological hazards associated with the raw materials employed in ‘Nduja production.

In light of these considerations, the present study aimed to evaluate, through experimental contamination study (controlled challenge tests), the behavior of *Salmonella* Typhimurium, *E. coli* O157:H7 and *L. monocytogenes* during the production process of ‘Nduja. By monitoring the evolution of these pathogens across manufacturing, this work contributes to the microbiological characterization of this traditional product, providing novel data of relevance for the scientific community, food business operators and competent authorities and ultimately supporting evidence-based approaches to its safe production and valorization.

## 2. Materials and Methods

### 2.1. Bacterial Strains and Inoculum Preparation

The following reference strains were used in the challenge test: *Salmonella enterica* subsp. *enterica* ser. Typhimurium ATCC 14028 (Genbank accession number DQ023313.1), *E. coli* O157:H7 CCUG29188 and *L. monocytogenes* ATCC 13932(Genbank accessione number CP025219.1). Working stocks were maintained at –80 °C in cryovials containing brain heart infusion (Biolife, Milan, Italy) supplemented with 15% glycerol (*v*/*v*) (Carlo Erba Reagents S.r.l., Milan, Italy) as cryoprotectant.

Prior to the challenge test, cryovials were thawed at refrigerate temperature and an aliquot of each strain was streaked onto Nutrient Agar (Scharlab, Lodi, Italy) using a 10 µL sterile loop. The plates were then incubated at 37 °C for 24 h.

A single well-isolated colony from each plate was then transferred into the corresponding liquid medium, Tryptone Soy Broth (Biolife, Milan, Italy) for *Salmonella* Typhimurium and *E. coli* O157:H7, and Tryptone Soy Yeast Extract Broth (Biolife, Milan, Italy) for *L. monocytogenes*, and incubated at 37 °C until reaching the stationary growth phase, as confirmed by a turbidity equivalent to 0.5 McFarland standard (approximately 10^8^ CFU/mL).

The broth cultures were then subjected to ten-fold serial dilutions in fresh medium until a final concentration of approximately 10^3^ CFU/mL was achieved. The diluted suspensions were subsequently incubated at 15 °C until reaching the stationary growth phase, as determined by comparison with a 0.5 McFarland standard, in order to allow bacterial adaptation to the temperature conditions applied during the simulated ‘Nduja production process [1]. The broth cultures obtained under these conditions were subsequently used for the experimental inoculation of ‘Nduja samples.

### 2.2. Experimental Production of ‘Nduja and Challenge Test Design

The experimental design of the challenge test carried out in the present study was inspired by the principles outlined in ISO 20976-2 [9].

The ‘Nduja samples used in this study were experimentally produced at the Pilot Plant of Food Technologies of the Department of Veterinary Sciences, University of Messina (Messina, Italy). Raw materials consisting of a pre-mixed blend of throat fat and lard were supplied by a local meat processing company located in Spilinga area (Vibo Valentia, Italy). At the pilot plant, commercial chopped semi-dried chili pepper (*Capsicum* spp.; Sapori di Sicilia, Vibo Valentia, Italy) and sodium chloride (Sapori di Sicilia, Vibo Valentia, Italy) were added to the raw material blend in proportions consistent with the traditional recipe [1] (approximately 25% *w*/*w* chili pepper and 3% *w*/*w* NaCl), and the mixture was then thoroughly homogenized using a paddle mixer.

Prior to experimental inoculation, a representative sample of the raw mixture was collected and analysed for the presence of the target pathogens (*Salmonella* Typhimurium, *E. coli* O157:H7 and *L. monocytogenes*), as described in Section 2.3.

The mixture was then experimentally inoculated with the broth cultures of the three target pathogens and thoroughly homogenized in the mechanical mixer to ensure an even distribution of the inoculum. The volume of inoculum added to the mixture was adjusted according to the concentration of each broth culture (approximately 10^8^–10^9^ CFU/mL), with the aim of achieving an initial contamination level of at least 10^7^ CFU/g.

Subsequently, the raw mixture was manually stuffed using a manual stuffer into glued natural pork casings and tied with twine to obtain six individuals ‘Nduja units, each weighing approximately 500 g.

In parallel, non-inoculated products (controls) were produced under the same conditions to verify that the inoculum did not result in significant differences in pH and a_w_ as described in Section 2.4.

The samples were initially stored for one week under progressively changing conditions, with decreasing temperature (from 20 °C to 15 °C) and increasing relative humidity (from 60% to 80%). Thereafter, the products were ripened at approximately 15 °C and 80% relative humidity. The pathogens were simultaneously co-inoculated into the ‘nduja to more accurately simulate a realistic contamination setting reflective of natural multi-pathogen exposure conditions.

Contaminated samples were then analysed for microbiological and physicochemical parameters as described in the following sections.

### 2.3. Microbiological Analysis

For the detection of *Salmonella* Typhimurium, *E. coli* O157:H7 and *L. monocytogenes* in the raw mixture before inoculation, three 25 g aliquots were aseptically collected.

The first aliquot (~25 g) was diluted with buffered peptone water (Biolife, Milan, Italy) at a ratio of 1:9 *w*/*v*, and then homogenized with a stomacher (400 Circulator; International PBI s.p.a., Milan, Italy) for 60 s at 230 rpm, for the detection of *Salmonella* spp. according to ISO 6579-1:2017 [10], with enrichment in Mueller Kauffmann Tetrathionate Broth base (Biolife, Milano, Italy) and Rappaport Vassiliadis Soy Broth (Biolife, Milano, Italy), followed by a smear on plates of Chromogenic Salmonella Agar (CSAB; Biolife, Milano, Italy) and Xylose Lysine Deoxycholate Agar (XLD agar; Biolife, Milano, Italy), both incubated at 37 ± 1 °C for 24 ± 3 h.

The second aliquot (~25 g) was diluted (ratio of 1:9 *w*/*v*) in modified Tryptone Soya Broth (Biolife, Milano, Italy) supplemented with 20 mg/L of novobiocin and incubated at 41.5 ± 1 °C for 18–24 h to detect *E. coli* O157:H7. One millilitre aliquot of the pre-enriched sample was added to a 20 µL Dynabead anti-*E. coli* O157 suspension (Thermo Fisher Scientific Inc., Monza, Italy), following the manufacturer’s instructions. The immunoconcentrated sample was then inoculated onto Cefixime-Tellurite Sorbitol MacConkey (CT-SMAC) agar (Biolife, Milan, Italy) and Chromogenic *E. coli* O157 agar (Biolife, Milan, Italy), and both were incubated for 18–24 h at 37 ± 1 °C.

The third aliquot (~25 g) was processed for the *L. monocytogenes* detection according to ISO 11290-1:2017 [11]. It involves dilution in Half-Fraser Broth Base (HFBB) (Biolife, Milan, Italy) at a ratio of 1:9 *w*/*v*, followed by homogenisation. The resulting homogenate was incubated at 30 ± 1 °C for 24 ± 1 h, followed by passage in Fraser Broth Base (FBB) (Biolife, Milan, Italy) with incubation at 37 ± 1 °C for 24 ± 1 h. The first (from HFBB) and the second enrichment (from FBB) were seeded onto Listeria Agar plates according to “Ottaviani and Agosti” (Biolife, Milan, Italy) and onto Listeria Palcam Agar (Biolife, Milan, Italy), both incubated at 37 ± 1 °C for 48 ± 2 h.

With regard to experimentally inoculated ‘Nduja units, sampling was performed at days 0, 7, 14, 21 and 28. At each sampling time point, triplicate samples were collected by removing approximately 100 g of product from three different units. The samples were subsequently divided for microbiological analyses and for the determination of physicochemical parameters (pH and a_w_).

For the enumeration of the investigated microbiological parameters, sample preparation was performed as described above using peptone saline water (Biolife, Milan, Italy) as diluent. Serial ten-fold dilutions were then prepared in the same diluent and used for microbiological enumeration. The following microbiological parameters were investigated: (i) enumeration of *Salmonella* Typhimurium plates of CSAB and XLD agar, both incubated at 37 °C for 24 h; (ii) enumeration of *E*. *coli* O157:H7 on plates of CT-SMAC and Chromogenic *E. coli* O157 agar both incubated for 18–24 h at 37 ± 1 °C; (iii) enumeration of *L. monocytogenes* according to ISO 11290-2:2017 [12] on plates of Agar Listeria according to Ottaviani and Agosti (Biolife, Milano, Italy) and Listeria Palcam Agar (Biolife, Milano, Italy), both incubated at 37 °C for 48 h; (iv) enumeration of Enterobacteriaceae, according to ISO 21528-2:2017 [13], on plates of Violet Red Bile Glucose Agar (Biolife, Milano, Italy) incubated at 37 °C for 24 h; (v) enumeration of yeasts and moulds according to ISO 21527-2:2008 [14] on plates of Dichloran-glycerol (DG18) agar base incubated at 25 °C for 120 h and (vi) enumeration of lactic acid bacteria according to ISO 15214:1998 [15] on plates of De Man, Rogosa and Sharpe (MRS) agar incubated at 30 °C for 72 h.

The detection limit for all enumeration methods was set at 10 CFU/g for Enterobacteriaceae and lactic acid bacteria and at 100 CFU/g for *Salmonella* Typhimurium, *E. coli* O157:H7, *L. monocytogenes*, yeasts and moulds. For modelling purposes, counts below the analytical detection limit were expressed as 0 log CFU/g to enable numerical processing and kinetic fitting of censored microbiological data. This conventional data treatment should not be interpreted as evidence of absolute microbial absence, but rather as an operational approach for handling non-quantifiable values. From an interpretative perspective, this approach remains conceptually comparable to alternative representations based on the analytical detection threshold, since the microbiological significance of the result remains unchanged, namely that microbial populations were below the quantification capability of the analytical method applied. All data are expressed as mean ± standard deviation of the three independent replicates.

### 2.4. pH and a_w_ Determination

At each sampling time point, pH and a_w_ measured in both the experimentally inoculated samples and control samples. The pH was measured by direct probe insertion using a calibrated pH meter with penetration electrode suitable for semi-solid foods (SevenDirect™ SD20, Mettler-Toledo, Greifensee, Switzerland). The a_w_ was determined using a calibrated dew-point water activity meter (Aqualab 4TE Duo, Steroglass, San Martino, Italy) at 25 °C in accordance with ISO 18787:2017 [16].

All data are expressed as mean ± standard deviation of the three independent replicates.

### 2.5. Mathematical Modelling of Inactivation Kinetics

The inactivation kinetics of the three target pathogens (*Salmonella* Typhimurium, *E. coli* O157:H7 and *L. monocytogenes*) during the production process were characterised through mathematical modelling. Given that quantitative data on the behaviour of these microorganisms in this specific product matrix are currently limited in the scientific literature, modelling was performed with the dual aim of describing the observed survival dynamics and providing primary kinetic parameters that may serve as a basis for future quantitative microbial risk assessment (QMRA) or process validation studies.

Mean log CFU/g values calculated from triplicate measurements at each sampling time point were used as input data for model fitting. Four primary inactivation models were applied: (i) the linear (log-linear) model, based on classical first-order inactivation kinetics [17,18]; (ii) the Weibull model in the Mafart formulation [19], which accommodates concave or convex survival curves through the shape parameter p; (iii) the biphasic model [20], which describes inactivation as the superposition of two sub-populations with distinct inactivation rates; and (iv) the Geeraerd shoulder–tail model [21], which explicitly accounts for a physiological shoulder phase prior to inactivation and a residual resistant tail.

All analyses were performed in Python (version 3.11.9; Python Software Foundation, Wilmington, DE, USA) using the SciPy scientific computing library (version 1.13.0; [22]. Model fitting was carried out via non-linear least squares regression (scipy.optimize.curve_fit function) for the linear and Weibull models, and via global optimisation based on the differential evolution algorithm (scipy.optimize.differential_evolution function) [23] for the biphasic and Geeraerd models, owing to the multimodal nature of their respective objective functions and the risk of convergence to local minima with gradient-based methods.

Goodness-of-fit was assessed using the coefficient of determination (R^2^), root mean square error (RMSE), Akaike Information Criterion (AIC) [24], and Bayesian Information Criterion (BIC) [25]. Model selection was based on minimisation of AIC and BIC combined with maximisation of R^2^, prioritising parsimony where comparable fits were observed.

## 3. Results

### 3.1. Microbiological Results

The experimental contamination study monitored the survival dynamics of three artificially inoculated pathogenic microorganisms and the background microflora during the 28-day aging process of ‘Nduja. Control samples tested negative for *Salmonella* Typhimurium, *E. coli* O157:H7 and *L. monocytogenes*, confirming the absence of these pathogens in the raw mixture before experimental contamination.

Among the experimentally added pathogens, *Salmonella* Typhimurium declined from an initial inoculation level of 7.10 ± 0.03 log CFU/g to values below the detection limit by day 28, with rapid decline already evident by day 7 (5.17 ± 0.12 log CFU/g), corresponding to a calculated reduction of 7.10 log CFU/g. However, since values below the analytical detection limit were treated as 0 log CFU/g for modelling purposes, the estimated reduction should be interpreted cautiously and may slightly overestimate the actual decrease. *E. coli* O157:H7 showed progressive decline from 5.48 ± 0.09 log CFU/g to 1.87 ± 0.15 log CFU/g at day 28, with notable reduction by day 7 (4.11 ± 0.07 log CFU/g), resulting in a total reduction of 3.61 log CFU/g. *L. monocytogenes* demonstrated the highest resistance among tested pathogens, declining from 4.36 ± 0.40 log CFU/g initially to 3.04 ± 0.07 log CFU/g at day 28, achieving only a limited reduction of 1.32 log CFU/g.

The background microflora exhibited distinct patterns during aging. Enterobacteriaceae declined from 7.68 ± 0.02 log CFU/g to levels below the analytical detection limit by the end of ripening. As values below the detection limit were treated as 0 log CFU/g for descriptive purposes, the actual extent of reduction may be slightly overestimated. Lactic acid bacteria increased from 5.27 ± 0.23 log CFU/g to 7.51 ± 0.65 log CFU/g, reaching peak levels by day 7 (7.24 ± 0.51 log CFU/g) and maintaining high counts throughout aging (Δ = +2.24 log CFU/g). Molds showed gradual decline from 2.81 ± 0.13 log CFU/g to 1.49 ± 0.50 log CFU/g (Δ = 1.32 log CFU/g), while yeasts remained relatively stable with minimal change from 4.39 ± 0.36 log CFU/g to 4.14 ± 0.14 log CFU/g, reaching maximum levels at day 21 (5.64 ± 0.30 log CFU/g) before declining (Δ = 0.25 log CFU/g).

**Figure 1:**
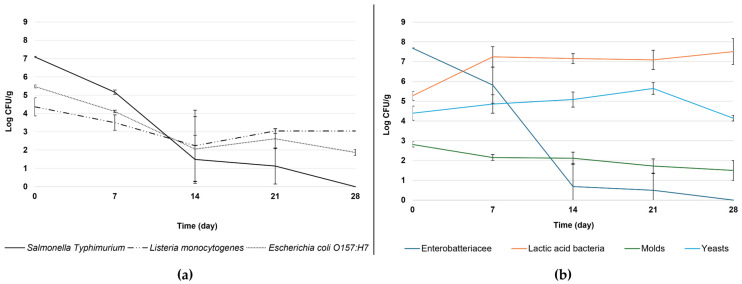
Survival dynamics of experimentally inoculated pathogens and evolution of background microflora during the 28-day aging of ‘Nduja. (**a**) Changes in the counts (log CFU/g) of *Salmonella* Typhimurium, *Escherichia coli* O157:H7 and *Listeria monocytogenes* in artificially inoculated samples. (**b**) Changes in background microflora, including Enterobacteriaceae, lactic acid bacteria, molds and yeasts, over the same period. Values are expressed as mean ± standard deviation.

The temporal trends of both pathogens and background microbiota are illustrated in Figure 1.

### 3.2. Physicochemical Parameters: pH and Water Activity

The evolution of pH and a_w_ was monitored throughout the 28-day ripening period in both the experimentally inoculated samples and the non-inoculated control products, produced in parallel under identical conditions with the aim of assessing whether the experimental inoculation exerted any measurable influence on the physicochemical dynamics of the ripening process.

In both sample groups, pH showed a progressive and consistent decline throughout the ripening period, reflecting the fermentative activity of the indigenous lactic acid bacteria microflora. In the inoculated samples, mean pH decreased from 4.77 ± 0.01 at day 0 to 4.14 ± 0.06 at day 28, with the most marked reduction observed between day 0 and day 7 (4.40 ± 0.30). A closely parallel trend was observed in control samples, where mean pH declined from 4.80 ± 0.11 at day 0 to 4.13 ± 0.08 at day 28. The two groups exhibited substantially overlapping values at all sampling time points, with differences in mean pH never exceeding 0.06 units across the entire observation period.

Water activity declined progressively in both groups throughout ripening, consistent with the progressive dehydration of the product matrix. In the inoculated samples, mean a_w_ decreased from 0.910 ± 0.001 at day 0 to 0.855 ± 0.019 at day 28, while control samples showed a reduction from 0.914 ± 0.005 to 0.855 ± 0.018 over the same period. The a_w_ values of inoculated and control samples were virtually indistinguishable at all time points, with mean differences ranging from 0.001 to 0.014 units and largely overlapping standard deviations throughout the monitoring period.

**Figure 2:**
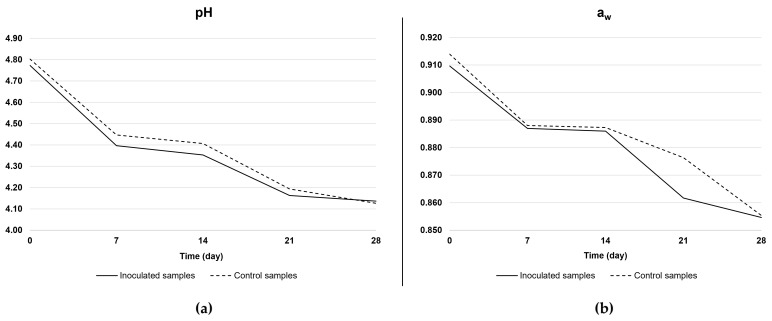
Temporal evolution of pH and water activity (a_w_) during the 28-day aging of ‘Nduja in experimentally inoculated samples with *Salmonella* Typhimurium, *Escherichia coli* O157:H7, and *Listeria monocytogenes*, and in control samples. (**a**) Changes in pH values. (**b**) Changes in water activity (a_w_). Values are expressed as mean of three independent replicates.

The temporal trends of pH and a_w_ in both inoculated and control samples are illustrated in Figure 2.

### 3.3. Mathematical Modelling of Inactivation Kinetics

The survival data obtained for the three inoculated pathogens were fitted to four primary inactivation models, linear (log-linear), Weibull, biphasic, and Geeraerd (shoulder–tail) [22]. Model parameters and goodness-of-fit statistics (R^2^, RMSE, AIC, BIC) for all pathogens are reported in Table 1.

For *Salmonella* Typhimurium, model performance improved progressively from the linear to the non-linear models. The biphasic model achieved the best overall fit (R^2^ = 0.978; AIC = –1.26), outperforming both the Weibull (R^2^ = 0.946; AIC = 1.27) and the Geeraerd model (R^2^ = 0.974; AIC = –0.31), while the linear model yielded the lowest goodness-of-fit (R^2^ = 0.917; AIC = 1.47). The best-fitting biphasic model estimated a D_1_-value of 2.52 days for the dominant sensitive fraction and a D_2_-value of 8.54 days for the resistant subpopulation, with a log N_res_ approaching the detection limit (0.55 log CFU/g) in the Geeraerd model.

For *E. coli* O157:H7, a similar ranking was observed, with non-linear models consistently outperforming the linear approach. The Geeraerd model achieved the best fit (R^2^ = 0.956; AIC = –4.47), followed by the biphasic (R^2^ = 0.947; AIC = –3.48), Weibull (R^2^ = 0.893; AIC = –1.98), and linear models (R^2^ = 0.804; AIC = –0.96). Notably, the Geeraerd model estimated a shoulder length of S_l_ = 1.69 days and a residual population of log N_res_ = 2.15 log CFU/g, consistent with the persistent tail observed in the experimental data.

For *L. monocytogenes*, all models produced substantially lower goodness-of-fit values compared to the other two pathogens. The Geeraerd model nonetheless achieved the highest performance among the tested models (R^2^ = 0.800; AIC = –3.69), followed by the biphasic (R^2^ = 0.780; AIC = –3.22), Weibull (R^2^ = 0.683; AIC = –3.39), and linear models (R^2^ = 0.400; AIC = –2.20). The estimated log N_res_ of 2.78 log CFU/g in the Geeraerd model and the D_2_ → ∞ in the biphasic model both indicate the presence of a subpopulation that was not inactivated throughout the 28-day ripening period.

Overall, considering all goodness-of-fit indices, the Geeraerd model provided the best fit for *E. coli* O157:H7 and *L. monocytogenes*, while the biphasic model achieved the highest performance for *Salmonella* Typhimurium. Across all three pathogens, the superiority of non-linear models over the log-linear approach was consistent, with AIC and BIC values systematically lower for the Weibull, biphasic, and Geeraerd models in all cases.

## 4. Discussion

The present study investigated the behaviour of *Salmonella* Typhimurium, *E. coli* O157:H7 and *L. monocytogenes* during the ripening of ‘Nduja, a traditional Calabrian spreadable fermented sausage for which quantitative microbiological data remain largely scarce in the scientific literature. To our knowledge, this represents one of the first challenge tests conducted directly in this product matrix, providing novel kinetic data on pathogen inactivation under conditions closely simulating artisanal production. The results demonstrate that the ripening process exerted a substantial, although heterogeneous, antimicrobial effect on the investigated pathogens. Salmonella Typhimurium declined to levels below the analytical detection limit by day 28. In accordance with the adopted treatment of censored microbiological data, values below the detection threshold were expressed as 0 log CFU/g exclusively for numerical processing and kinetic modelling purposes. This conventional approach does not imply absolute microbial absence and does not affect the overall biological interpretation of the observed pathogen behaviour during ripening, since values below the detection limit consistently indicate non-quantifiable microbial populations under the applied analytical conditions. In contrast, E. coli O157 showed a reduction of 3.61 log CFU/g, while L. monocytogenes exhibited a markedly limited decrease of only 1.32 log CFU/g over the entire observation period. The initial inoculum levels recorded at day 0 were lower than the nominal target of 10^7^ CFU/g for *L. monocytogenes* (4.36 ± 0.49 log CFU/g) and *E. coli* O157:H7 (5.48 ± 0.09 log CFU/g). These discrepancies are attributable, at least in part, to the cold-stress adaptation step included in the inoculum preparation protocol, whereby broth cultures were maintained at 15 °C following initial growth at 37 °C. Although this step was intentionally designed to acclimate bacteria to the ripening temperature conditions, consistently with ISO 20976-2 [9], it inevitably imposed suboptimal growth conditions, particularly for the strictly mesophilic *E. coli* O157:H7, whose proliferation at 15 °C is inherently limited and associated with cold-shock responses and potentially reduced colony-forming efficiency [26,27]. Since the primary aim of the challenge test was to characterize inactivation kinetics rather than to compare absolute residual counts across pathogens, these differences were considered acceptable and methodologically consistent with comparable studies on fermented meat products [28,29]. Regarding the simultaneous inoculation of three pathogens as a mixed culture, while inter-species competition is theoretically a confounding factor, its practical significance is likely marginal in the present study. The target organisms collectively represented a minor fraction of the total microbial load relative to the dominant indigenous LAB population, which reached 7.24 ± 0.51 log CFU/g by day 7 and was maintained throughout ripening. The antimicrobial pressure exerted by LAB-driven fermentation far outweighs any inter-pathogen competitive effect, and the differential inactivation profiles observed are more plausibly explained by the distinct intrinsic stress tolerances of each organism [30]. The progressive inactivation of all three pathogens was driven by the convergence of multiple antimicrobial hurdles acting simultaneously on the product matrix, consistently with the hurdle technology framework [30]. Among these, LAB proliferation played a central role by promoting progressive acidification of the product. The pH data clearly documented this dynamic: mean pH declined from 4.77 ± 0.01 at day 0 to 4.14 ± 0.06 at day 28 in inoculated samples, with a virtually identical trend in controls (4.80 ± 0.11 to 4.13 ± 0.08), confirming that experimental inoculation did not alter the fermentation dynamics of the product. Notably, the already markedly acidic pH at day 0 (~4.77–4.80) reflects the intrinsic acidity contributed by the chili pepper component and the inhibition of endogenous alkalizing microflora from the earliest stages of production [31]. In parallel, mean a_w_ declined from 0.910 ± 0.001 to 0.855 ± 0.019 in inoculated samples and from 0.914 ± 0.005 to 0.855 ± 0.018 in controls, with negligible differences between the two groups at all time points, driven by the combined effects of NaCl addition, progressive dehydration and the inherently low moisture content of the high-fat matrix. The synergistic action of reduced pH and a_w_ is well documented in fermented meat products [30,32]. An additional antimicrobial contribution may derive from capsaicin and related capsaicinoids from chili pepper (25% *w*/*w*), though their effective aqueous-phase concentration is likely reduced by partitioning into the abundant lipid fraction of the matrix [33]. The three pathogens exhibited markedly different responses to these combined hurdles, with a resistance hierarchy of *L. monocytogenes* > *E. coli* O157:H7 > *Salmonella* Typhimurium, broadly consistent with findings reported for other fermented meat products [29,32]. Salmonella Typhimurium demonstrated the greatest susceptibility, declining to levels below the analytical detection limit by day 28 and exhibiting biphasic reduction kinetics with D1 = 2.52 days and D2 = 8.54 days, suggesting phenotypic heterogeneity within the population and differential responses to the cumulative stresses associated with ripening [34,35]. *E. coli* O157:H7 showed intermediate susceptibility, with a 3.61 log CFU/g reduction and a persistent residual population of ~2.15 log CFU/g at day 28. The initial shoulder (S_1_ = 1.69 days) identified by the Geeraerd model, during which minimal inactivation occurred despite the already acidic matrix pH, is consistent with the activation of the well-characterised acid tolerance response (ATR) of this pathogen, renowned for its exceptional acid resistance [36]. The persistence of a residual population above the detection limit throughout the ripening period raises food safety concerns, particularly given the very low reported infectious dose of *E. coli* O157:H7 (10–100 CFU) [37]. From a food safety perspective, the behaviour of *L. monocytogenes* requires a balanced interpretation. Although a transient increase was observed at intermediate stages of ripening, no net growth occurred over the entire process. By the end of the 28-day period, counts were overall stable or slightly reduced (−1.32 log CFU/g), indicating that the product environment was not conducive to sustained proliferation and exerted a certain inhibitory effect. However, this reduction remained limited, and the convergent prediction of both the Geeraerd (log N_res_ = 2.78 log CFU/g) and biphasic (D2 → ∞) models suggests the persistence of a residual subpopulation effectively refractory to complete inactivation throughout the ripening period. Several mechanisms may contribute to this exceptional persistence [38]. The psychrotrophic character of *L. monocytogenes* and its capacity to express broad-spectrum stress response regulons, including the master regulator σ^B^, governing acid, osmotic, and oxidative stress resistance [39], at 15 °C confer a substantial advantage over the other two mesophilic pathogens [40]. Furthermore, the extremely high lipid content of ‘Nduja (~50% adipose tissue) may exert a direct protective effect: bacteria may become physically associated with lipid droplets within the matrix, shielding them from the aqueous phase where antimicrobial compounds exert their activity [41]. The hydrophobic surface properties of *L. monocytogenes*, attributable to its teichoic acid-rich cell wall, may further promote such lipid-associated microenvironments [42], a mechanism particularly relevant in ‘Nduja given its fat content far exceeding that of conventional dry-fermented sausages. Finally, the systematic superiority of non-linear inactivation models over the log-linear approach, consistent across all three pathogens, confirms that microbial inactivation in this complex matrix cannot be adequately described by first-order kinetics. The observed shoulders, concavities, and persistent tails reflect genuine biological phenomena including phenotypic heterogeneity, adaptive stress responses, and the differential resistance of subpopulations occupying distinct microenvironments within the food matrix [43,44]. The particularly poor fit of the log-linear model to *L. monocytogenes* data (R^2^ = 0.400) is mechanistically coherent with its essentially flat survival curve. These results reinforce the recommendation that model selection in challenge test analysis should be based on systematic goodness-of-fit assessment across multiple primary models [45]. The kinetic parameters generated, D-values, shape parameters, shoulder lengths, and tail levels for all three pathogens, constitute novel primary inactivation data for ‘Nduja that may serve as a quantitative basis for future QMRA applications and process validation studies for this traditional product. From a food safety perspective, the results may contribute to quantitative microbial risk assessment (QMRA) and process validation frameworks for fermented meat products. The pathogen-specific reduction patterns highlight the contribution of combined intrinsic hurdles in limiting pathogen survival during ripening. However, the persistence of *L. monocytogenes* and residual levels of *E. coli* O157 throughout the experimental period indicates that these findings should be interpreted as quantitative evidence of pathogen behaviour during ripening rather than as definitive evidence of product safety. Overall, these data may support HACCP-based hazard evaluation and the refinement of predictive models for traditional high-fat fermented sausages such as ‘Nduja. Therefore, the present findings should be interpreted as quantitative evidence of pathogen behaviour during ripening rather than as definitive evidence of product safety.

## 5. Conclusions

The present study provides, for the first time, quantitative data on the inactivation kinetics of *Salmonella* Typhimurium, *E. coli* O157:H7 and *L. monocytogenes* during the ripening of ’Nduja, contributing to the microbiological characterization of this traditional product, for which scientific evidence remains limited. The results demonstrate that the combined antimicrobial hurdles associated with the ripening process, including LAB-driven acidification, progressive reduction of water activity, and the intrinsic physicochemical properties of the matrix, exert a strong inhibitory effect on all three pathogens. In particular, *Salmonella* Typhimurium was declined to levels below the detection limit within 28 days; therefore, the calculated reduction should be interpreted conservatively, since values below the detection limit were assigned to 0 log CFU/g for modelling purposes. For *E. coli* O157:H7 and *L. monocytogenes*, a transient increase was observed at intermediate time points; however, no overall growth occurred throughout the ripening period. At the end of ripening, *E. coli* O157:H7 exhibited a slight reduction in viable counts, whereas *L. monocytogenes* remained stable overall, indicating that the product environment did not support sustained pathogen proliferation under the tested conditions. Under the tested experimental conditions, the physicochemical characteristics of the product appeared unfavorable for sustained pathogen proliferation; however, microbial persistence patterns differed among the investigated microorganisms. Finally, the superiority of non-linear inactivation models over the classical log-linear approach, consistently observed across all three pathogens, further highlights the inadequacy of first-order kinetic assumptions in describing pathogen behavior in complex fermented meat matrices. The kinetic parameters generated in this study represent a novel and valuable dataset that may support future quantitative microbial risk assessment and evidence-based process validation for this traditional Calabrian product.

## Figures and Tables

**Table 1:** Primary parameters of inactivation models fitted to mean log CFU/g data for *Salmonella* Typhimurium, *Listeria monocytogenes*, and *Escherichia coli* O157:H7 during 28-day ripening of ‘Nduja.

Pathogen	Model	log N_0_ (log CFU/g)	Primary Parameters	R^2^	RMSE	AIC	BIC
*Salmonella* Typhimurium	Linear	6.62	k = 0.261 day^−1^; D = 3.84 days	0.917	0.776	1.47	0.69
	Weibull	7.22	α = 1.50 days; β = 0.689; δ = 5.04 days	0.946	0.623	1.27	0.10
	Biphasic ★	7.34	f = 1.000; k1 = 0.397 day^−1^; D_1_ = 2.52 days k_2_ = 0.117 day^−1^; D_2_ = 8.54 days	0.978	0.396	−1.26	−2.83
	Geeraerd	7.60	k = 0.963 day^−1^; S_l_ = 1.19 days; log N_res_ = 0.55	0.974	0.436	−0.31	−1.88
*Listeria monocytogenes*	Linear	3.86	k = 0.044 day^−1^; D = 22.53 days	0.400	0.538	−2.20	−2.98
	Weibull	4.37	α = 1.76 days; β = 0.157; δ = 352.5 days	0.683	0.391	−3.39	−4.56
	Biphasic	4.43	f = 0.977; k1 = 0.180 day^−1^; D_1_ = 5.55 days k_2_ ≈ 0; D_2_ → ∞	0.780	0.326	−3.22	−4.78
	Geeraerd ★	4.86	k = 0.521 day^−1^; S_l_ = 2.21 days; log N_res_ = 2.78	0.800	0.311	−3.69	−5.25
*Escherichia coli *O157:H7	Linear	4.97	k = 0.124 day^−1^; D = 8.04 days	0.804	0.609	−0.96	−1.74
	Weibull	5.51	α = 1.99 days; β = 0.496; δ = 10.69 days	0.893	0.450	−1.98	−3.15
	Biphasic	5.61	f = 0.999; k_1_ = 0.257 day^−1^; D_1_ = 3.89 days k_2_ = 0.010 day^−1^; D_2_ = 100.5 days	0.947	0.317	−3.48	−5.04
	Geeraerd ★	5.98	k = 0.676 day^−1^; S_l_ = 1.69 days; log N_res_ = 2.15	0.956	0.287	−4.47	−6.04

log N_0_: initial pathogen concentration (log CFU/g) estimated by the model at time t = 0. k: maximum inactivation rate constant (day^−1^). In the linear model, k is the slope of the log-linear decline. D: decimal reduction time (days) in the linear model; time required for a 1 log^10^ reduction in population (D = 1/k). α: Weibull scale parameter (days); β: Weibull shape parameter (dimensionless). β < 1 indicates concave (tailing) kinetics; β > 1 indicates convex (shouldering) kinetics; β = 1 is equivalent to the linear model. δ: Weibull time for the first 1 log^10^ reduction, analogous to the D-value [δ = α (ln 10)^1/β^]. f: fraction of the sensitive subpopulation in the biphasic model (0–1); k_1_, D_1_: inactivation rate constant and decimal reduction time of the sensitive fraction; k_2_, D_2_: inactivation rate constant and decimal reduction time of the resistant fraction. S_l_: shoulder length (days) in the Geeraerd model; duration of the initial lag phase before exponential inactivation begins. log N_res_: log concentration of the residual (tail) subpopulation that is not inactivated over the observation period (log CFU/g). R^2^: coefficient of determination; RMSE: root mean square error; AIC: Akaike Information Criterion; BIC: Bayesian Information Criterion. Lower AIC and BIC values and higher R^2^ indicate better model fit. ★ Best-fitting model for each pathogen, selected on the basis of lowest AIC and BIC and highest R^2^.

## Data Availability

The original contributions presented in this study are included in the article. Further inquiries can be directed to the corresponding author.

## Abbreviations

The following abbreviations are used in this manuscript:

LAB	Lactic acid bacteria
CSAB	Chromogenic Salmonella Agar
CT-SMAC	Cefixime-Tellurite Sorbitol MacConkey
HFBB	Half-Fraser Broth Base
FBB	Fraser Broth Base
AIC	Akaike Information Criterion
R^2^	Coefficient of determination
BIC	Bayesian Information Criterion
RMSE	Root mean square error
XLD agar	Xylose Lysine Deoxycholate Agar
